# The Influence of Education on Chinese Version of Montreal Cognitive Assessment in Detecting Amnesic Mild Cognitive Impairment among Older People in a Beijing Rural Community

**DOI:** 10.1155/2014/689456

**Published:** 2014-05-28

**Authors:** Shu'aijun Zhou, Jianzhong Zhu, Na Zhang, Bailing Wang, Tao Li, Xiaozhen Lv, Tze Pin Ng, Xin Yu, Huali Wang

**Affiliations:** ^1^Dementia Care & Research Center, Peking University Institute of Mental Health (Sixth Hospital), Key Laboratory for Mental Health, Ministry of Health, No. 51 Huayuanbei Road, Beijing 100191, China; ^2^Wuxi Mental Health Center, Wuxi 214151, China; ^3^Beijing Anzhen Hospital, Capital University of Medical Sciences, Beijing 100029, China; ^4^Qingdao Mental Health Center, Qingdao 266034, China; ^5^Department of Psychological Medicine, National University of Singapore, Singapore 117597

## Abstract

To assess the influence of education on the performance of Chinese version of Montreal cognitive assessment (C-MoCA) in relation to the mini-mental state examination (MMSE) in detecting amnesic mild cognitive impairment (aMCI) among rural-dwelling older people C-MoCA and MMSE was administered and diagnostic interviews were conducted among community-dwelling elderly in two villages in Beijing. The performance of C-MoCA and MMSE in detecting aMCI was evaluated by the area under the ROC curve (AUC). Effect size of education on variations in C-MoCA scores was estimated with general linear model. Among 172 study participants (24 cases of aMCI and 148 normal controls), the AUC of C-MoCA was 0.72 (95% CI = 0.62–0.81, cutoff = 20/21), compared to AUC of MMSE of 0.74 (95% CI = 0.64–0.84, cutoff = 26/27). The performance of both C-MoCA and MMSE was especially poorer among those with low (0–6 years) education. After controlling for gender and age, education (**η** ^2^ = 0.204) had a surpassing effect over aMCI diagnosis (**η** ^2^ = 0.052) on variations in C-MoCA scores. Among rural older people, the MoCA showed modest accuracy and was no better than MMSE in detecting aMCI, especially in those with low education, due to the overwhelming effect of education relative to aMCI diagnosis on variations in C-MoCA performance.

## 1. Introduction

It is estimated that 44 million people worldwide are currently living with dementia, with the numbers doubling every 20 years [1]. As the world's most populous country with the largest aging population, China faces an extraordinary challenge of Alzheimer's disease (AD) and other age-related cognitive disorders. Currently a crucial area of research and clinical interest with the focus on secondary prevention of dementia is the detection of early AD and mild cognitive impairment (MCI).

MCI is a prodromal syndrome of AD and dementia characterised as cognitive impairment beyond normal ageing, with minimal or no decline in activities of daily living (ADL). It appears that approximately 10% to 15% of the subjects of MCI evolve to AD each year [2]. The mini-mental state examination (MMSE) is widely used as a brief cognitive screening instrument for the detection of dementia but has limited sensitivity in detecting early cognitive impairment. The Montreal cognitive assessment (MoCA) was developed as a brief screening instrument for MCI and mild Alzheimer disease (AD) to address the known limitations of the MMSE [3]. According to earlier studies, the MoCA has shown excellent performance in discriminating aMCI from normal cognition among old people, with reported AUCs ranging between 0.82 and 0.95, sensitivity from 78% to 97%, and specificity from 60% to 87% [3–6]. Most of these earlier studies have evaluated the MoCA among urban-dwelling elderly with generally better education in socioeconomically developed countries.

Cognitive performance as measured by the MMSE is known to be influenced by education [7], and appropriate cutoffs are frequently employed to adjust for differences in educational levels. An emerging number of studies especially of populations living in less developed economies increasingly suggest that MoCA is also influenced by education level, thus requiring varying cutoffs to be employed because of cultural and language differences [4, 8–15]. However, few of these studies provided estimates of the effect sizes due to MCI relative to education or systematically evaluated the MoCA's test performance separately for better and less educated groups. At the same time, a limited amount of data have emerged to suggest that the MoCA may not show good test performance at least in some subpopulations [12].

To date, few studies have evaluated the MoCA's test performance among rural people. Over 38% of the population of China lives in rural areas, most of whom are lowly educated. We thus investigated the performance of the Chinese version of MoCA (C-MoCA) in detecting aMCI among elderly persons in a rural community, a majority of whom have low level of education. We evaluated the relative effect sizes of education and aMCI on variations in the MoCA scores and compared their test performance among those with less (0–6 years) education and those with more (7–12 years) education. At the same time, the MMSE was evaluated in the same manner for comparison.

## 2. Methods

### 2.1. Subjects

Individuals with aMCI and cognitively healthy individuals were recruited from participants in a cross-sectional study designed to assess common psychological problems of Chinese older persons. This study was supported by the projects in the National Science and Technology Pillar Program during the Eleventh Five-Year Plan Period. The target sample size was 300 and the actual sample size was 360 considering the 20% nonresponse rate. A total of 360 community-dwelling elderly aged ≥60 years were randomly sampled from two villages in Haidian District, Beijing. The villages are not far from each other with similar folk customs and environmental conditions. Two hundred and five individuals (response rate: 56.9%) completed the interview and neuropsychological assessment and were independently diagnosed as aMCI or cognitively normal. A total of 172 cases (24 cases of aMCI and 148 cognitively normal controls) were included in this analysis. All participants provided informed written consent. This study was approved by local ethical committee.

### 2.2. Diagnostic Criteria

Clinical diagnostic interviews were conducted by trained psychiatrists and diagnosis of aMCI was made according to the methods described previously [16]. Briefly, the operational diagnostic criteria of aMCI were defined as follows: (1) the global cognitive function was in keeping with normal cognition, (2) the patient complained of memory deterioration, or the relatives and doctors thought the patient had memory impairment, and the duration of the symptom was less than 3 months, (3) the ability of daily life and social function was declining; the total score of activities of daily living (ADL) was not more than 18, (4) the rank score of global deterioration scale (GDS) was 2 to 3, (5) the score of clinical dementia rating (CDR) was 0.5, (6) the score of Auditory Verbal Learning Test of World Health Organization-battery of cognitive assessment instrument for elderly (WHO-BCAI-AVLT) was no more than mean score −1.5 SD, (7) did not meet DSM-IV criteria for the diagnosis of dementia, and (8) Hachinski ischemic score (HIS) was less than 4.

The exclusion criteria were as follows: (1) mixed dementia and vascular dementia; the HIS was not less than 4, (2) other neurodegenerative disease, such as Parkinson's disease (PD), frontotemporal dementia (FTD), dementia with Lewy bodies (DLB), cognitive dysfunction and dementia induced by the traumatic brain injuries, tumor, and infection, (3) cognitive dysfunction induced by the endocrine system disease such as thyroid hypofunction and adrenocortical insufficiency, (4) cognitive dysfunction and dementia induced by the serious diseases related to cardiovascular, respiratory, hepatic, renal, or hematopoietic system, (5) cognitive dysfunction and dementia induced by epilepsy, (6) cognitive dysfunction and dementia induced by alcohol, drug, and psychotropic drugs, and (7) cognitive dysfunction and dementia induced by other physical and chemical factors.

The criteria of normal control (NC) were defined as follows: (1) cognitive function was normal, (2) no serious physical diseases, and (3) could cooperate to complete relevant examination.

### 2.3. Neuropsychological Assessment

The battery of neuropsychological tests conducted by trained psychologists included MMSE and C-MoCA.

**Figure 1:**
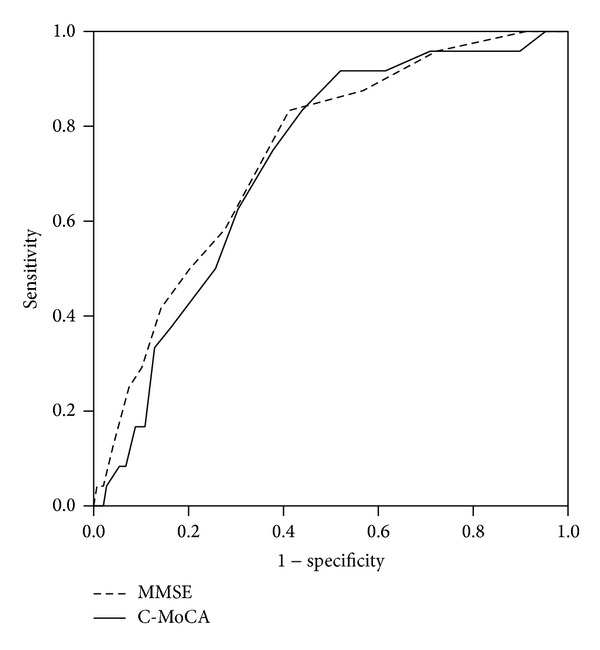
Receiver operating characteristic curve of MMSE and C-MoCA. The ROC curves were used to detect the amnesic MCI. C-MoCA: the Chinese version of Montreal cognitive assessment. MMSE: mini-mental state examination.

The MMSE is a widely used, standardized screening test used to evaluate general cognitive functioning and takes less than 20 minutes to complete. MMSE has a maximum score of 30 points involving different cognitive domains: orientation of three words (3 point), subtracting serial sevens from 100 (5 points), language testing by naming objects (2 points), repeating a sentence (1 point), and comprehension tested by complying with a three-step command (3 points) and copying a spatially arranged design of figures (1 point) [17].

The MoCA measures eight cognitive domains on a single page, which are scored within a range of 0–30 points (higher scores indicating better function). The items are visuospatial abilities and executive function (cube drawing: 1 point, clock drawing: 3 points, trail-making test: 1 point), naming (3 points), attention, concentration, and working memory (digit span: 2 points, cancelation: 1 point; subtraction: 3 points), sentence repetition (2 points), verbal fluency (1 point), abstract ability (2 points), short-term memory (delayed recall: 5 points), and orientation (time orientation: 3 points and space orientation: 3 points). The C-MoCA used in the study is available at http://www.mocatest.org. Items in the C-MoCA are identical to the English version of MoCA with the exception of several cultural and linguistic modifications.

### 2.4. Statistical Analyses

**Figure 2:**
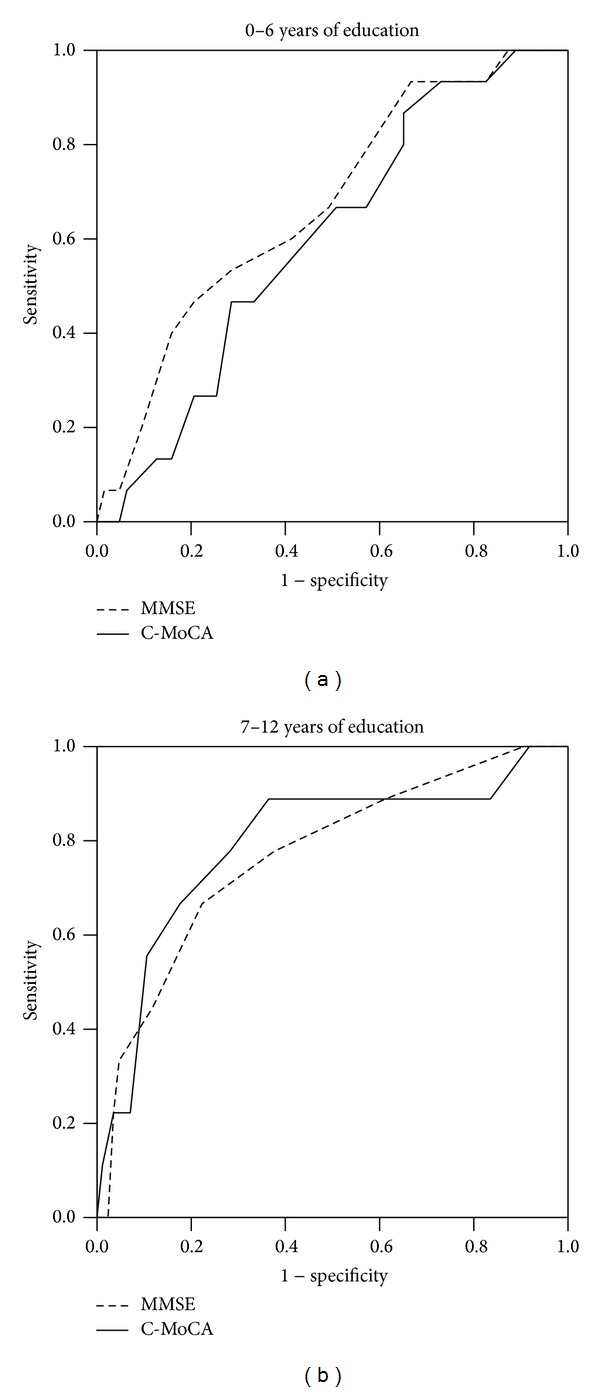
Receiver operating characteristic curve of MMSE and C-MoCA in different education groups. The ROC curves were used to detect the amnesic MCI for different education groups: (a) for 0–6 years of education group and (b) for 7–12 years of education group. C-MoCA: the Chinese version of Montreal cognitive assessment. MMSE: mini-mental state examination.

ROC curve analysis was used to evaluate the performance of MMSE and MoCA in discriminating aMCI from cognitive healthy controls. Independent* t*-test, chi-square test, and rank sum test were used to analyze the differences between two educational groups (primary education group: 0–6 years and secondary education group: 7–12 years) (Figure 2). The relative effect sizes of age, gender, education, and aMCI on variations in C-MoCA scores were estimated from analysis of variance in general linear model. Variations of C-MoCA and MMSE scores were assessed by partial eta squared (*η* ^2^). SPSS for Windows version 17.0 was used for statistical analyses.

## 3. Results

### 3.1. Demographic Characteristics

There were no significant differences between aMCI group and NC group in age, education, physical job, smoking, and drinking; only women were proportionately more in the aMCI group (*P* < 0.05, Table 1). However, there were no gender differences in performance scores on the C-MoCA. The mean C-MoCA scores (adjusted for age, education, and aMCI diagnosis) in men (20.6 ± 0.53) were not significantly different from those in women (19.7 ± 0.42, Table 2).

### 3.2. Variations of C-MoCA Scores by Education and aMCI Status

Table 3 shows the regression estimates of the independent associations of gender, age, education, and aMCI status with C-MoCA and MMSE scores. Similar patterns of relationships with gender, age, education, and aMCI status were observed for both C-MoCA and MMSE scores. There were significant differences in mean scores of C-MoCA by educational groups and aMCI status. The adjusted mean C-MoCA score associated with 0–6 years of education was significantly lower by 3.7 points (*P* < 0.001) than that with 7–12 years of education. The mean scores of C-MoCA were significantly lower by 2.5 points in the aMCI group than in the control group (*P* < 0.001, Table 2). Notably, lower education showed considerably larger partial eta squared (*η* ^2^ = 0.204) to variations in C-MoCA scores than aMCI diagnosis (*η* ^2^ = 0.052). Thus, while the individual variables except for gender were significantly independent predictors of C-MoCA scores, they differ in their effect sizes.

### 3.3. ROC Curve of MMSE and C-MoCA

As shown in Table 1 and Figure 1, overall, the AUC of C-MoCA in discriminating between aMCI and cognitively normal controls was 0.72 (95% CI: 0.62–0.81), with optimal sensitivity of 0.75 and specificity of 0.62 using a cutoff of 20/21. In comparison, the AUC of MMSE was 0.74 (95% CI: 0.64–0.84), with optimal sensitivity of 0.83 and specificity of 0.56 using a cutoff of 26/27. There was no significant difference in AUC's between the C-MoCA and MMSE.

### 3.4. C-MoCA and MMSE ROCs by Educational Groups

Table 3 shows the mean scores and test performance parameters of C-MoCA and MMSE scores by educational groups. As expected, within each educational stratum, the mean C-MoCA and MMSE scores were significantly lower in aMCI participants than in NC participants except for C-MoCA mean scores in the 0–6 years educational group. In this group, there was only a 1.4 point difference in mean C-MoCA scores between aMCI and NC participants, which was not statistically significant.

Test performance of the C-MoCA was better in the 7–12 years educational group (AUC = 0.79; sensitivity of 0.89 and specificity of 0.64 at cutoff 22/23) and poorer in the 0–6 year educational group (AUC = 0.60; sensitivity of 67% and specificity of 49% at cutoff 18/19). In comparison, C-MoCA did not show better test performance characteristics than the MMSE. While its test performance was marginally better than the MMSE in the better educated group, it was marginally poorer for the low level educational group.

## 4. Discussion

In a large majority of previously published reports, the C-MoCA has shown high detection accuracy, sensitivity, and specificity at various cutoffs for detecting MCI, including in patients performing in the normal range on the MMSE [3]. Our evaluation among community-dwelling elderly with generally lower education in two rural villages in China, however, showed that the AUC (0.72) of the C-MoCA is lower than in previous studies, which generally reported AUC's in the 0.80s or 0.90s. Most previously published studies evaluating the MoCA have either been conducted on urban-dwellers or did not report urban-rural differences. For example, our results may be contrasted with the high AUC (0.88), sensitivity (77%), and specificity (90%) reported among elderly urban-dwellers in Shanghai [18].

Our findings are supported by preliminary data from two recent studies. An earlier study of the MoCA reported modest accuracy (AUC: 0.71 with sensitivity of 68.7% and specificity 63.9% at a lowered cutoff score 22 [14]. This is a study in Beijing involving a large heterogeneous population sample which includes the sample of rural elderly persons in this study. However, data in another study of Singapore Chinese elderly persons also indirectly show modest accuracy of MoCA for MCI detection (AUC < 0.77), sensitivity of 96% but specificity of 30% at cutoff of 25/26. Furthermore, we found that the C-MoCA appeared not to have an advantage over MMSE in discriminating between aMCI and normals. This concurs with the previously published study in north China [14].

Our results showed lower mean C-MoCA's test scores associated with low education, confirming observations from an increasing number of studies that education influences the C-MoCA's test scores. Estimates of the influence of education on MoCA scores and its heterogeneity of effect in different populations are not well documented. Crude comparisons of published data suggest that these estimates of education effect varied across different populations. In this study, there was a (unadjusted) 5-point difference in mean C-MoCA score between low and higher education groups (Table 4). This is comparable to the mean score of MoCA in a Portuguese population [4], which was approximately 4 points lower for primary (less than 6 years) education compared to higher educated groups, whereas the Los Angeles Chinese study showed only a 2.6-point difference in MoCA scores between 1–6 years and 7–11 years education groups [15]. These comparisons, however, do not take into account population differences in age and the great heterogeneity in population characteristics across studies. More studies of the effect of education vis-à-vis MCI diagnosis on variations in MoCA scores and their heterogeneity of effects across different populations should be conducted.

Various studies have determined different MoCA cutoffs in discriminating MCI from normal but mostly without educational stratification [8–15]. A minority of studies that reported educational stratifications of MoCA cutoffs determined lower MoCA cutoffs for elderly persons with low education but with wide differentials in cutoff points. For example, in the Los Angeles Chinese study, this was lowered by 2 points from 22/23 for middle level education (7–11 years) to 20/21 for low level education (0–6 years) [15]. In our study, we lowered the differential by 4 points from 22/23 for higher (7–12 years) education to 18/19 for low (0–6 years) education. Another Chinese population study determined even wider differential of MoCA cutoffs, from 24/25 for 7 or more years of education to 19/20 for 1–6 years of education and 13/14 for no education [10].

The observed wide variations in MoCA test performance across different studies may be due to linguistic and cultural differences between the original English version and indigenous versions of the MoCA, the lower education level of populations in developing countries, and the strong effect of education on MoCA scores. Previous studies do not provide sufficient information to assess the relative effect of education vis-à-vis MCI diagnosis on MoCA test scores and the influence of education on MoCA test performance. Our study showed that in multivariate analysis controlling for gender and age, low education (0–6 years) was associated with an adjusted 3.6-point lower C-MoCA score whereas aMCI diagnosis was associated with a 2.4-point lower C-MoCA score. Uniquely, our data thus indicated that education had an overwhelmingly surpassing effect than MCI diagnosis on C-MoCA test scores, at least among rural elderly persons. Further studies should be conducted in heterogeneous population samples to document the relative effect of education and MCI diagnosis.

**Figure 3:**
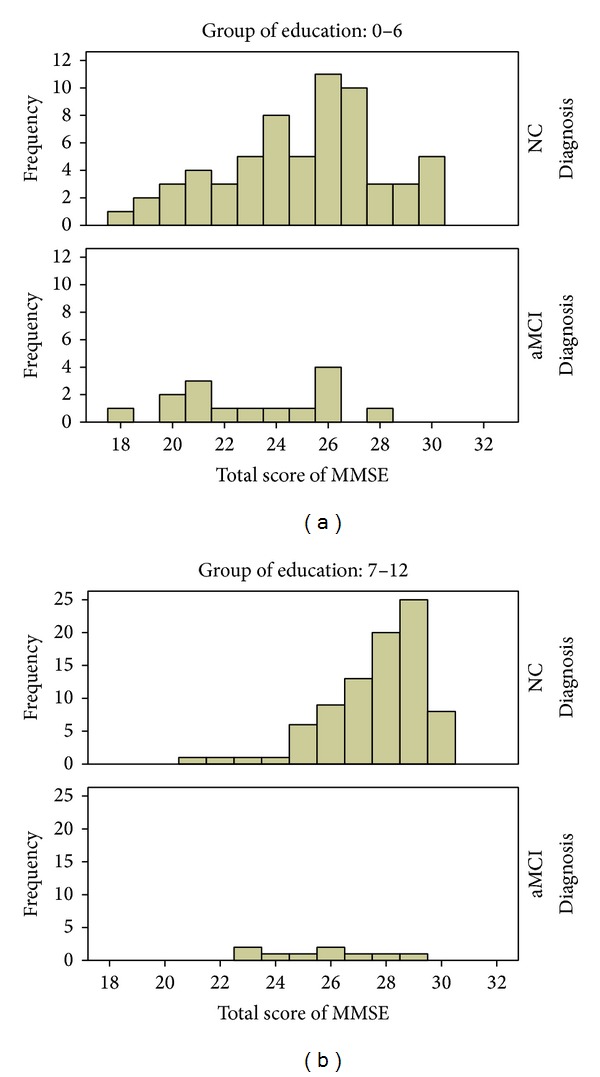
Frequency distribution of C-MoCA scores by aMCI status and education groups. (a) The 0–6 years of education group; (b) the 7–12 years of education group. NC: the participants for normal control; aMCI: the participants for amnesic mild cognitive impairment.

It would appear that the MoCA was designed to be free of the ceiling effect to improve on the MMSE to detect mild cognitive impairment and early dementia. It does this by increasing difficulty in the item responses (increasing failure rates) thus shifting response scores toward lower values to the left, at the same time making the distribution normal (Figure 3). However, this may have increased the tendency for greater floor effects for low cognitive functioning individuals, including those with MCI as well as less educated individuals. It would appear, therefore, that, in populations with large proportions of poorly educated individuals, the low cognitive functioning of MCI due to an underlying brain pathology becomes indistinct with the low cognitive functioning associated with low education. This happens when low cognitive functioning associated with low education is more profound in some population groups such as rural people with poorer quality education.

For practical purposes, the Chinese version of MoCA appears to be satisfactory for detecting MCI among better educated Chinese elderly persons, as shown in this study (AUC = 0.79), as well as in previous studies of the Chinese MoCA in urban elderly populations in Shanghai and Beijing [13, 18]. There is grave concern about using the C-MoCA among less educated Chinese elderly, at least those living in rural villages, given the serious problems of low power of detection and missed diagnoses. More research is needed to develop cognitive screening tools that are universally appropriate for all population groups including rural people.

Our study had several limitations. The sample size was small. Hence, these results may be viewed as only preliminary, pending further larger studies. Another limitation is that the study used secondary data from surveys in the National Science and Technology Pillar Program designed to assess common psychological problems of Chinese older persons. The primary research objective was not to evaluate the C-MoCA for detecting amnesic mild cognitive impairment. The C-MoCA and MMSE were among a large number of scales used, and psychological pressure and fatigue could possibly influence the detection utility of the C-MoCA, although they were administered first in line with other scales.

## 5. Conclusion

The Chinese version of MoCA has modest accuracy and is not better than the MMSE in detecting mild cognitive impairment especially among rural-dwelling Chinese older persons with low education. Education exerts a stronger influence than MCI diagnosis on variations in MoCA scores, and this is likely to adversely affect its test performance among lowly educated individuals.

## Figures and Tables

**Table 1:** Demographic characteristics of participants and mean C-MoCA and MMSE.

	Amnesic MCI (*N* = 24)	Normal control (*N* = 148)	*P* value
Age, mean (SD)	67.17 ± 6.59	67.66 ± 7.16	0.752
Female, *N* (%)	19 (79.2%)	83 (56.1%)	0.043
Education, mean (SD)	6.17 ± 1.24	6.96 ± 0.49	0.230
Manual job, *N* (%)	22 (91.7%)	136 (91.9%)	0.611
Current smoking, *N* (%)	4 (16.7%)	53 (35.8%)	0.100
Current alcohol drinking, *N* (%)	3 (12.5%)	39 (26.4%)	0.201
MMSE, mean ± SD	24.1 ± 1.22	26.5 ± 0.44	0.000
C-MoCA, mean ± SD	18.3 ± 1.56	21.5 ± 0.73	0.001
C-MoCA			
AUC	0.72 ± 0.10		
Cutoff	20/21		
Sensitivity	0.75		
Specificity	0.62		
MMSE			
AUC	0.74 ± 0.10		
Cutoff	26/27		
Sensitivity	0.83		
Specificity	0.56		

The *P* values were calculated using Student's *t*-test for continuous variables and chi-square test for categorical variables.

C-MoCA: the Chinese version of Montreal cognitive assessment.

MMSE: mini-mental state examination.

ROC curve: receiver operating characteristic curve.

AUC: area under receiver operating characteristic curve.

**Table 2:** Unadjusted and adjusted mean C-MoCA and MMSE scores by gender, education, and aMCI diagnosis.

	*N*	C-MoCA		MMSE	
		Unadjusted mean (SD)	Adjusted mean ± SE	Unadjusted mean (SD)	Adjusted mean ± SE
Gender					
Male	70	21.5 (4.1)	21.6 ± 0.53	26.4 (2.4)	25.6 ± 0.35
Female	102	20.7 (4.7)	19.7 ± 0.42	26.0 (3.1)	25.2 ± 0.28
Education					
Less (0–6 years)	78	18.5 (4.4)	18.3 ± 0.47	24.6 (3.1)	24.3 ± 0.31
More (7–12 years)	94	23.2 (3.4)	21.9 ± 0.48	27.5 (1.9)	26.5 ± 0.32
Amnesic MCI					
Yes	24	18.3 (3.7)	18.9 ± 0.72	24.1 (2.9)	24.4 ± 0.48
No	148	21.5 (4.5)	21.3 ± 0.29	26.5 (2.7)	26.4 ± 0.19

C-MoCA: the Chinese version of Montreal cognitive assessment.

MMSE: mini-mental state examination.

Amnesic MCI: amnesic mild cognitive impairment.

**Table 3:** Regression estimates of relative contributions of age, education, and aMCI diagnosis to C-MoCA scores.

	C-MoCA					MMSE				
	Regression coefficient	SE	*t*	*P*	*η* ^2^	Regression coefficient	SE	*t*	*P*	*η* ^2^
Male (versus female)	0.91	0.55	1.647	0.101	0.016	0.36	0.36	0.995	0.32	0.006
Age, per year	−0.23	0.04	−5.871	0.0001	0.171	−0.13	0.03	−5.199	0.0001	0.136
0–6 years education (versus 7–12 years)	−3.61	0.55	−6.536	0.0001	0.204	−2.13	0.36	−5.837	0.0001	0.169
Amnesic MCI (versus normal control)	−2.34	0.77	−3.014	0.003	0.052	−1.99	0.51	−3.894	0.0001	0.083

**Table 4:** C-MoCA and MMSE of aMCI and normal controls and test performance by educational groups.

	Less (0–6 years) education			More (7–12 years) education		
	Amnesic MCI (*n* = 15)	Normal control (*n* = 63)	*P*	Amnesic MCI (*n* = 9)	Normal control (*n* = 85)	*P*
C-MoCA, mean (SD)	17.3 ± 2.04	18.7 ± 1.15	0.266	20.0 + 2.46	23.52 ± 0.68	0.002
MMSE, mean (SD)	23.1 ± 1.63	25.0 ± 0.76	0.039	25.7 ± 1.63	27.6 ± 0.39	0.003
C-MoCA						
AUC ± SE	0.60 ± 0.14			0.79 ± 0.17		
Cutoff	18/19			22/23		
Sensitivity	0.67			0.89		
Specificity	0.49			0.64		
MMSE						
AUC ± SE	0.67 ± 0.14			0.77 ± 0.16		
Cutoff	25/26			27/28		
Sensitivity	0.67			0.78		
Specificity	0.51			0.62		

The *P* values were calculated using Student's *t*-test for continuous variables.

AUC: area under receiver operating characteristic curve.
